# Acceptability measures of water, sanitation and hygiene interventions in low- and middle-income countries, a systematic review

**DOI:** 10.1371/journal.pntd.0010702

**Published:** 2022-09-12

**Authors:** Rose Hosking, Suji Y O’Connor, Kinley Wangdi, Johanna Kurscheid, Aparna Lal

**Affiliations:** 1 National Centre for Epidemiology and Population Health, College of Health and Medicine, Australian National University, Acton, Australia; 2 Swiss Tropical and Public Health Institute, Associate Institute of University of Basel, Allschwil, Switzerland; McGill University Faculty of Medicine and Health Sciences, CANADA

## Abstract

**Background:**

Inadequate access to water, sanitation, and hygiene (WASH) is an environmental risk factor for poor health outcomes globally, particularly for children in low- and middle-income countries (LMIC). Despite technological advancements, many interventions aimed at improving WASH access return less than optimal results on long term impact, efficacy and sustainability. Research focus in the ‘WASH sector’ has recently expanded from investigating ‘which interventions work’ to ‘how they are best implemented’. The ‘acceptability’ of an intervention is a key component of implementation that can influence initial uptake and sustained use. Acceptability assessments are increasingly common for health interventions in clinical settings. A broad scale assessment of how acceptability has been measured in the WASH sector, however, has not yet been conducted.

**Methods/Principal findings:**

We conducted a systematic literature review of intervention studies published between 1990 and 2021 that evaluated the acceptability of WASH interventions in LMIC settings. Using an implementation science approach, focused outcomes included how acceptability was measured and defined, and the timing of acceptability assessment. We conducted quality assessment for all included studies using the Cochrane Risk of Bias tool for randomised studies, and the Newcastle-Ottawa Scale for non-randomised studies.

Of the 1238 records; 36 studies were included for the analysis, 22 of which were non-randomized interventions and 16 randomized or cluster-randomized trials. We found that among the 36 studies, four explicitly defined their acceptability measure, and six used a behavioural framework to inform their acceptability study design. There were few acceptability evaluations in schools and healthcare facilities. While all studies reported measuring WASH acceptability, the measures were often not comparable or described.

**Conclusions:**

As focus in WASH research shifts towards implementation, a consistent approach to including, defining, and measuring acceptability is needed.

## Author summary

The COVID-19 pandemic highlighted the need for sustainable and effective water, sanitation and hygiene (WASH) interventions in low and middle-income countries (LMIC). There is a significant body of literature measuring the impact of WASH interventions on health outcomes, such as diarrhoea or soil-transmitted helminth infections. The importance of behavioural determinants is also recognized and measured. However, many WASH interventions are not sustainable long-term. To improve this, research focus in the WASH sector has recently shifted towards implementation. This includes a significant number of process evaluations that focus on how well programs are implemented.

A key component of implementation that has been recognized for health interventions in clinical settings is the initial and ongoing ‘acceptability’ to the recipient population. Sekhon et al. developed a theoretical framework of acceptability to capture the aspects of acceptability that may influence intervention uptake and sustained use. This is the first review of acceptability of WASH interventions. The outcomes of this review add new evidence on how and why it has been measured, and the potential place of acceptability evaluations in WASH to improve the impact and sustainability of interventions. The findings of this review may be useful for WASH stakeholders including intervention researchers, implementers, and recipients.

## Introduction

Inadequate access to water, sanitation, and hygiene (WASH) is an environmental risk factor for poor health outcomes globally, particularly for children in low- and middle-income countries (LMIC) [1]. Adverse health outcomes associated with poor WASH include worm, enteric, respiratory, skin and ear infections [2]. Despite technological advancements, we remain short of achieving universal access to ‘safe’ WASH by 2030; goal 6 of the United Nations Sustainable Development Goals [3]. WASH interventions, such as infrastructure provision (e.g. taps, latrines, soap), and education programs aimed at behaviour change are critical for achieving this goal and have been implemented widely in low-resource settings [4]. However, many interventions return less than optimal results on long term impact, efficacy and sustainability [5].

A significant body of WASH intervention research focuses on which interventions ‘work’. This is commonly determined by changes in health or behavioural outcomes [6,7]. Examples of health metrics include diarrheal incidence, worm infections [6,7], treatment outcomes and growth rates in children [8]. Changes in WASH-related behaviour, and attitudes towards a health outcome are also frequently assessed through self-reported ‘knowledge, attitudes and practice’ surveys [9]. Other measured outcomes include patient satisfaction [10], absenteeism and cognitive performance in children [8]. A framework for incorporation of behavioural determinants into WASH intervention design and evaluation has also been developed through systematic review [11].

To improve impact and sustainability, research focus in the ‘WASH sector’ has recently expanded from investigating ‘which interventions work’ to ‘how they are best implemented’ [12]. The increased focus on implementation has led to the promotion of community-based behaviour change approaches and the inclusion of psychosocial theory in WASH intervention delivery [9]. The ‘acceptability’ of an intervention is a key component of implementation [13]. This is because acceptability can influence initial uptake, and sustained use of an intervention [14]. Sekhon et al. recently developed the theoretical framework of acceptability (TFA) for healthcare interventions [15]. The TFA outlines seven component constructs of acceptability: “affective attitude, burden, perceived effectiveness, ethicality, intervention coherence, opportunity costs, and self-efficacy.” While acceptability assessments of healthcare interventions are increasingly common in clinical settings [15], their place in WASH has not yet been reviewed.

To address this gap, the aim of this review is to synthesise the existing literature on the acceptability measures of WASH interventions and methods of measurement in resource poor settings. We achieve this by an implementation science approach to address the following questions: (1) In what range of settings has the acceptability of WASH interventions been evaluated? (2) What methods have been used to evaluate acceptability? (3) How has acceptability been defined in these different contexts? In answering these questions, we make recommendations on the utility and current methods for evaluating the acceptability of WASH interventions.

## Methods

We designed the review in accordance with the Preferred Reporting Items for Systematic Reviews and Meta-Analyses (PRISMA) guidelines. Inclusion and exclusion criteria can be found in Table A in S1 Table.

### Search strategy

We searched online databases for WASH intervention studies that purported to measure acceptability published between January 1990 and December 2021. The search was restricted to articles published in the English language (Box 1).

**Box 1. Databases and search terms for the systematic review on acceptability of WASH interventions**

We searched PubMed, Web of Science, Scopus and the Cochrane Collaboration databases using the following key words in the title, abstract or topic:

(WASH OR “water, sanitation and hygiene” OR water OR sanitation OR hygiene OR toilet OR latrine OR handwash* OR “drinking water”) AND intervention AND (accept*)

### Study selection

One author (RH) removed duplicates and conducted the initial title scan. The title and abstracts were independently screened by two authors (RH, SYO). A full-text review of retained articles was then conducted by two authors (AL, KW). Any conflicts were resolved by discussion between four authors (AL, KW, RH, SYO). Forward and backward citation analysis and hand-search of reference lists were undertaken for all included studies, and relevant reviews and meta-analyses identified.

### Data analysis and quality assessment

Two authors (RH, SYO) extracted data using standardized extraction tables. The pro forma included acceptability definition, method of measurement, timing, use of the acceptability assessment and whether the study was a precursor to further randomised controlled trials (RCTs) or implementations. We undertook quality assessment for all included studies. The Cochrane risk-of-bias tool (ROB-2) was used for randomised studies [16]; degree of bias was determined using the pre-existing criteria set out by Cochrane. Non-randomised studies were assessed using an adapted version of the Newcastle-Ottawa Quality Assessment Scale (NOS) developed by Modesti and Colleagues [17]. NOS scores were considered as follows: 0–3 low quality, 4–6 moderate quality, and 7–10 high quality.

## Results

### Search results

**Fig 1:**
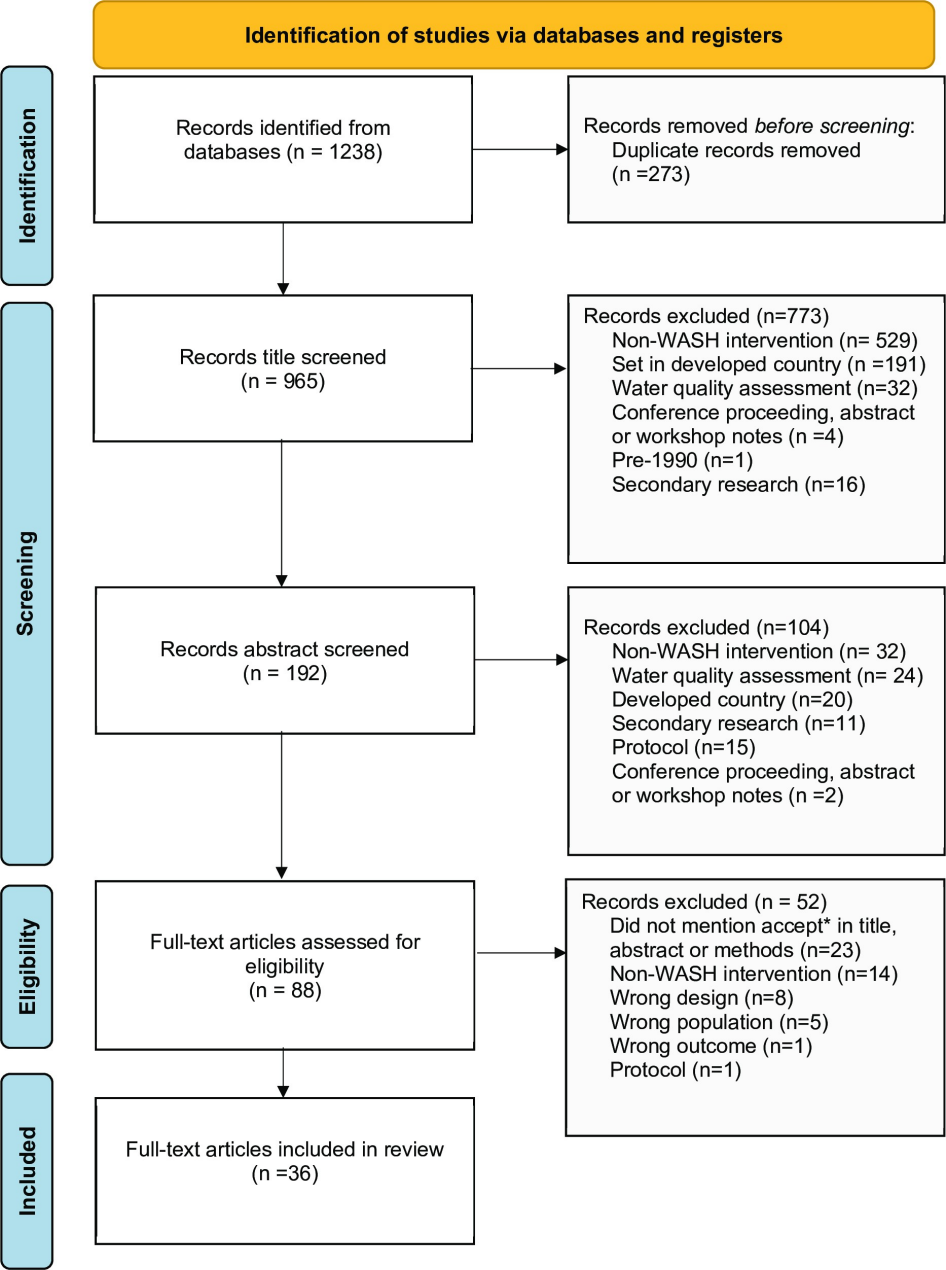
PRISMA diagram of search and included articles.

The initial search strategy generated 1238 articles, after removing duplicates and screening non-relevant abstracts, 87 full text articles were assessed for eligibility. Of the 87 studies included for full text review, 36 met the inclusion criteria (Fig 1).

### Designs, settings and WASH interventions

The majority (22/36) of identified studies were non-randomized designs including cross-sectional, case-control, household trials (aka trials of improved practice) and pilot and feasibility studies (Table 1). The remaining 14 studies were RCT or cluster-RCTs (Table 2). Of the included studies, 29 were household-level interventions. Most of these studies (22/29) were implemented in rural areas, but three of these also occurred in urban areas and seven in urban areas only. All household studies assessed physical interventions; in ten cases this was combined with a health education component. Five of the household studies also included a community-level intervention aimed at more than one household.

**Table 1:** Research articles published January 1990- December 2021 where the acceptability of water, sanitation or hygiene intervention was evaluated, by year–non-randomized designs.

Study	Design	Setting	Level	Intervention type	Intervention	Acceptability measure^	Measurement method	Timing	Quality
Aikhomu et al. (2000) [27]	Case-control	Rural	Households	Physical intervention	Communal water filtration units made from metal oil drums with a filter cloth inserted in the top and spigots at the bottom.	Perceptions of positive and negative features	Survey	Retrospective	Moderate
Rainey et al. (2005) [28]	Cross-sectional	Rural	Households	Physical intervention	Solar water disinfection (SODIS) in a village in Nepal.	Based on Health Belief Model	Survey	Retrospective	Moderate
Simms et al. (2005) [29]	Cross-sectional	Rural	Households	Physical intervention	Follow up study of improved pit latrines provided as part of a trachoma control programme in Gambia.	Satisfaction (happy/ unhappy)	Interview	Retrospective	Moderate
Rose et al. (2006) [30]	Cohort	Urban	Households	Physical intervention	100 children were assigned to receive drinking water subject to SODIS with 100 age and sex matched controls.	Feelings towards the intervention, ease, cost, limitations	Focus Group + Interview + Survey	Retrospective	Moderate
Diallo et al. (2007) [31]	Cross-sectional	Rural	Households	Health Education + Physical intervention	Installed latrines in Zinder, Niger & health education on personal hygiene and environmental sanitation.	Reported advantages of latrine use (vs. disadvantages)	Interview	Retrospective	Moderate
Hulland et al. (2013) [32]	Household trials	Urban + Rural	Households	Physical intervention	Assessment of seven candidate handwashing technologies during two iterative phases.	Satisfaction, willingness to use, and perceived appropriateness of handwashing station	Interview	Retrospective	High
Francis et al. (2015) [33]	Cross-sectional	Rural	Community	Physical intervention	The Skyhydrant, a high throughput membrane filter for drinking water installed in 5 kiosks in 3 villages.	Support for the intervention, willingness to pay for clean water	Focus group + Interview	Retrospective	Moderate
Hogarh et al. (2015) [34]	Cross-sectional	Rural	Households	Physical intervention	Point-of-use water filter, the ‘biosand filter’.	Willingness to purchase, interest	Interview	Prospective	Moderate
Kundu et al. (2016) [22]	Cross-sectional	Rural	Households + Community	Physical intervention	Three safe drinking water interventions: the arsenic removal household (Sono) filter, community deep tube well, and an improved dug well.	Authors’ definition of social acceptance,: “the willingness of users to receive and use a technology”.	Focus group + Interview	Retrospective	Moderate
Ashraf et al. (2017) [35]	Household trials	Rural	Households	Health Education + Physical intervention	Non-randomized trial of strategies to promote soapy water for handwashing. Three intervention arms: promotion only, promotion and handwashing stations and promotion, stations, and detergent refills.	Derived from IBM-WASH: convenience, ease of use, perceived value and sharing, motivations for use, experiences, and barriers.	Focus group + Interview	Retrospective	High
Hussain et al. (2017) [23]	Household trials	Rural	Households	Physical intervention	Three locally available child potty models.	“An acceptable behaviour … in which participants are willing to adopt and practice, that is feasible, practical, beneficial, and can be adjusted through negotiation”	Focus group + Interview	Prospective & Retrospective	Moderate
Yeasmin et al. (2017) [36]	Pilot & feasibility	Urban + Rural	Households + Community	Health Education + Physical intervention	Behaviour change communication discouraging rubbish disposal in communal toilets and installation and promotion of rubbish bins next to toilets.	Derived from IBM-WASH: perceptions, benefits and barriers.	Focus group + Interview	Prospective	High
Crider, et al. (2018) [37]	Cross-sectional	Urban	Households	Physical intervention	Drinking water chlorination, 25 tasted sodium hypochlorite and 25 tasted NaDCC.	Perceived taste acceptability threshold	Survey	Prospective	High
Sultana et al. (2018) [38]	Pilot & feasibility	Urban	Households	Health Education + Physical intervention	The ‘soapy water bottle’ handwashing system was introduced to households and promoted by community health promoters and a supervisor. Monthly meeting were held to educate about key handwashing times.	Derived from IBM-WASH: satisfaction	Focus group + Interview	Retrospective	Moderate
Yeasmin et al. (2019) [18]	Pilot & feasibility	Urban + Rural	Schools	Health Education + Physical intervention	A 1 month intervention in 4 schools consisting of POU drinking water hardware, teacher training on drinking chlorinated water, cue cards and visual aids.	None	Focus Group + Interview + Survey	Prospective & Retrospective	Moderate
Alam et al. (2020) [39]	Mixed-methods	Urban*	Households + Community	Physical intervention	Piped water chlorination program and household level chlorine tablet distribution	Barriers and motivations.	Focus group + Interview	Retrospective	Moderate
Bitew et al. (2020) [40]	Cross-sectional	Urban + Rural	Households	Physical intervention	SODIS was implemented in some villages in an earlier trial and control villages where it was not implemented.	Cultural acceptance, barriers and enablers	Focus group + Interview	Retrospective	Moderate
Campbell et al. (2020) [19]	Mixed-methods	Urban	Healthcare	Health Education	Visual reminder tools for Hand Hygiene and brief verbal instruction aimed at families in paediatric intensive care unit	None	Focus group + Survey	Prospective & Retrospective	Moderate
Guo et al. (2021) [41]	Qualitative	Rural	Households	Physical intervention	New sanitation chains in rural China.	Perceived social acceptability	Survey	Retrospective	Moderate
Sutherland et al. (2021) [42]	Mixed-methods	Urban	Households	Physical intervention	Autarky handwashing station, with an on-site water recycling system called the Water Wall (treats and recycles water).	Perceived social acceptability, feelings towards site	Focus group + Survey	Prospective & Retrospective	Moderate
Thorseth et al. (2021) [43]	Pilot & feasibility	Urban	Households	Physical intervention	Modified hygiene kits: including either additional liquid soap, scented soap bar or a mirror	Likes and dislikes, desirability, pleasantness, long lasting, familiarity, want, likeliness of use, effective, easy, water-saving	Focus group	Retrospective	High
Yeasmin et al. (2021) [44]	Pilot & feasibility	Rural	Households	Physical intervention	Pilot study of the acceptability and barriers to use of hand sanitiser, soap or soapy water for hand-washing.	Derived from IBM-WASH: participants’ use	Focus group + Interview	Prospective	High

^ The definition of acceptability is recorded with quotations ““if the definition was supplied by the authors. Otherwise, the definition is supplied by the reviewers.

*Newcastle-Ottowa Scale (low quality, moderate quality, high quality)

**Table 2:** Research articles published January 1990- December 2021 where the acceptability of water, sanitation or hygiene intervention was evaluated, by year–randomized designs.

Study	Design	Setting	Level	Intervention type	Intervention	Acceptability measure^^^	Measurement method	Timing	Risk of Bias*
Firth et al. (2010) [45]	RCT	Rural	Households	Health Education + Physical intervention	Hygiene education and 4 water-purification intervention arms: closed valve container, *M*. *oleifera seeds*, chlorine or control.	Satisfaction, interest, compliance, preference	Survey	Prospective & Retrospective	High
McGuigan et al. (2011) [46]	RCT	Rural	Households	Physical intervention	SODIS in the intervention arm and no treatment of drinking water in the control group.	Use after 6 months; taken to be culturally acceptable (compliance)	Interview	Retrospective	Some concerns
Habib et al. (2013) [47]	Cluster-RCT	Rural	Households + Community	Health Education + Physical intervention	Intervention group received a “diarrhoea pack” containing zinc tablets, water purification tablets and an education leaflet) via community health workers and the control group received existing health care provisions only.	Usage, perceived effectiveness, willingness to purchase	Survey	Retrospective	High
Rajaraman et al. (2014) [21]	RCT	Rural	Community	Health Education	Health promotion campaign of ‘SuperAmma’ targeted at children; included a cartoon, posters, rewards and certificates for children who pledged to practice hand washing with soap, community events.	“Things liked and not liked”	Interview	Retrospective	Some concerns
Biswas et al. (2017) [48]	RCT	Rural	Households	Health Education + Physical intervention	One group received a handwashing station, the other received a promotion encouraging self-creation of a handwashing station.	Motivations, ease of use, costs, barriers, likes/dislikes	Focus group + Interview	Retrospective	Some concerns
Biran et al. (2018) [49]	RCT	Urban	Community	Health Education	An intervention arm received an inclusiveness training workshop for community-led total sanitation facilitators to improve access to sanitation for people with disability, controls did not receive this inclusiveness training.	Whether it was offensive, willingness to do actions	Interview	Retrospective	High
Ditai et al. (2018) [50]	Pilot & feasibility	Rural	Households	Health Education + Physical intervention	Three different ABHR formulations: plain, bitterant and perfumed in 100mL bottles. Used for 5 days and followed by a 2 week ‘washout’ period.	Overall satisfaction	Survey	Retrospective	High
McGuiness et al. (2018) [20]	RCT	Rural	Households	Physical intervention	Sequential introduction of piped riverbank filtration-treated drinking water and control was initial delivery of piped untreated water. Hygiene and safe water storage education given before study commencement.	Based on COB-M to identify barriers and enablers	Focus group + Interview	Retrospective	Some concerns
Stone et al. (2018) [51]	Pilot & feasibility	Rural	Community	Health Education	Interactive DVD about basic hygiene intervention and control receiving no education.	Attendance at the health session	Observation	Retrospective	High
Harrison et al. (2019) [52]	Cluster-RCT	Rural	Households	Health Education	An educational poster “Newborn Moments for Hand Hygiene in the Home” (as part of the wider BabyRub Pilot study)	Participants’ understanding, compliance to actions on poster	Focus group	Retrospective	High
Rajasingham et al. (2019) [53]	Cluster-RCT	Urban	Community	Physical intervention	Supply of 8.68g sodium dichloroisocyanurate tablets for water treatment to community drinking water vendors.	Use of the water treatments, ease, perceptions of customer preferences	Focus group + Survey	Retrospective	Moderate
Heitzinger et al. (2020) [24]	RCT	Urban	Households	Physical intervention	Trialled feasibility and acceptability of two different water pasteurization indicators.	“Participant’s satisfaction with use of the models”	Interview	Prospective & Retrospective	Some concerns
Ngasala et al. (2020) [54]	RCT	Rural	Households	Physical intervention	Each home provided with either chlorine tablets, silver-infused ceramic tablets, or SODIS	Attitude towards the intervention	Survey	Retrospective	Some concerns
Budge et al. (2021) [55]	RCT	Rural	Households	Physical intervention	Introduction of a household play space for infants (protective, walled enclosure) to limit direct ingestion of soil and faeces and protect from contaminated surfaces.	Acceptability of use, acceptability of design, and time use	Focus group	Prospective	High

^ The definition of acceptability is recorded with quotations ““if the definition was supplied by the authors. Otherwise, the definition is supplied by the reviewers.

* Cochrane (low risk, some concerns, high risk)

Five studies evaluated the acceptability of a community-level intervention only. Three of these were ‘health education’ interventions involving community workshops and education sessions and two were community water interventions. We identified two WASH intervention studies that measured acceptability in schools or healthcare facilities. The school study evaluated a drinking water hardware and hygiene education intervention in rural and urban Bangladesh [18]. The healthcare study occurred in a Vietnamese paediatric intensive care unit and evaluated the acceptability of visual reminders for hand hygiene [19].

### Methods of acceptability evaluations

The prospective (i.e. anticipated) acceptability of the intervention was measured in 11 studies. Of these, six also measured retrospective (i.e. experienced) acceptability. All of the prospective evaluations used either focus group discussions, interviews or surveys (Tables 1 and 2). Prospective evaluation was used to make changes to the design, communication, or implementation of the WASH interventions for subsequent randomized controlled trials in seven cases. The remaining five studies made recommendations for changes to the intervention without further trialling. Three found the intervention to be acceptable and recommended wider implementation (Table A and B in S2 Table).

The remaining 27 studies evaluated retrospective acceptability. The timing of these evaluations ranged from one day to four years later (Table A and B in S2 Table). The most-common follow-up times were 3, 6 and 12 months after the intervention was initiated. Three of the studies that conducted retrospective evaluations were ‘pilot and feasibility studies’ undertaken in preparation for further randomized controlled trials or wider implementations of the evaluated intervention. Retrospective evaluation was used to make changes to, or select the best, intervention design for subsequent trials or implementation in five cases (Table A in S2 Table). One used a retrospective evaluation to explain the results of an RCT with low uptake and adherence [20]. The remaining studies used the evaluations to make recommendations for further use or changes to future implementations without specified subsequent trials or implementations.

### Defining acceptability

**Fig 2:**
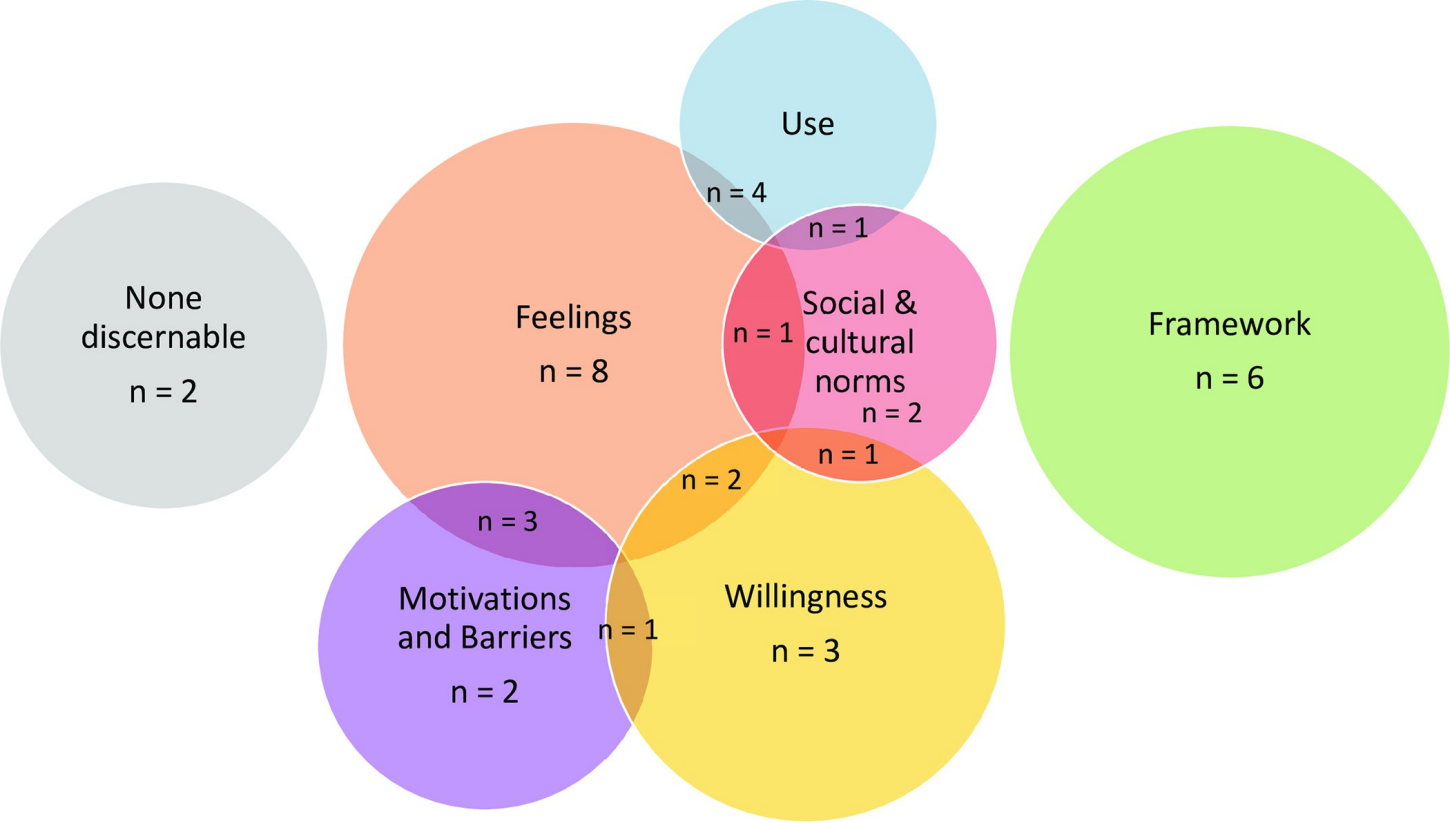
Definitions of acceptability of water, sanitation and hygiene interventions published 1990–2021 (including explicit and implicit definitions). Venn diagram is not to scale.

Across the 36 included studies, four explicitly defined their acceptability measure: ("things liked and not liked" [21]; "social acceptance, i.e., the willingness of users to receive and use a technology” [22]; “An acceptable behaviour is one in which participants are willing to adopt and practice, that is feasible, practical, beneficial, and can be adjusted through negotiation” [23]; and “participant’s satisfaction with use of the models” [24]). For the remaining studies, we extracted implicit definitions based on provided interview/survey results or terminology used by the authors. These fell into six distinct but overlapping groups: social/cultural acceptability, behavioural models, measures of use (uptake, compliance, adherence, and adoption), willingness (to use or purchase), barriers and motivations, and feelings towards the interventions (Fig 2).

Five studies based their evaluation on health behaviour models, four of which describe the Integrated Behavioural Model for water, sanitation and hygiene (IBM-WASH) [25]. The remaining study considered the Health Belief Model [26]. For explanation of reviewers grouping of implied definitions, see Table A in S4 Table.

### Risk of bias

All 14 randomised studies were found to be at risk of bias based on the Cochrane RoB assessment [16]. Seven were at high risk of bias, and seven had some concerns relating to bias. Among the 28 non-randomised studies, only four were classed as ‘high quality’ in accordance with the modified Newcastle-Ottawa Scale [17] (Table A, B and C in S3 Table).

## Discussion

There is a substantial gap in the inclusion of consistent measures of acceptability across WASH intervention studies. This review identified 36 articles evaluating the acceptability of WASH interventions in LMIC published between 1990 and 2021. These studies comprise a fraction of WASH interventions implemented in the same time period [9,12]. Given the significant amount of resources that have already been spent on WASH service delivery[56], and the amount required to reach universal access by 2030[57], it is important to maximise the effectiveness of WASH by considering acceptability as a key component that must be evaluated to increase sustainability[13].

There were few acceptability studies in schools and healthcare settings. While schools and healthcare facilities are priority targets for WASH interventions in LMIC [58], the lack of acceptability evaluations in these settings is stark. Infections due to inadequate WASH can spread quickly in these settings [59–61]. A lack of WASH effectiveness evaluations in health care facilities and schools was recently highlighted in a global evidence and gap map [9]. Acceptability evaluations that involve school children and healthcare workers in co-design have the potential to encourage hygiene and sanitation related behaviour at a large scale and drive sustainability of WASH interventions. Schools and healthcare facilities may also be less homogenous than single communities, so they are vital conduits to spread health promotion messages and increase adoption of healthy behaviours to diverse audiences [9].

Evaluating prospective acceptability is valuable because it can influence participation and uptake rates [14], and we recommend that this is routinely included in implementation. Only one-third of the WASH studies evaluated the prospective acceptability of the interventions; fewer still used this information to influence the design or implementation of the intervention. Prospective evaluation also encourages genuine community co-design which can further improve intervention effectiveness [62], and be used to refine intervention design prior to larger trials or wide implementation [15], ensuring the efficient use of resources. Most of the included studies conducted prospective assessments with a combination of focus group discussions and interviews.

Given the time and resource constraints commonly imposed on WASH service delivery [9], cross-sectional surveys with potential users may be more widely achievable. Electronic surveys are an increasing possibility as the number of internet and digital technology users in LMIC steadily climbs [63]. Retrospective acceptability evaluations can improve the sustained use of interventions, but their timing should be carefully considered, and based on whether the intervention is physical or sessional. Evaluations of physical interventions should allow sufficient time for users to experience any difficulties in use and maintenance without external support. Educational interventions that are sessional in nature, such as community education sessions or meetings, should be evaluated soon enough that participants have had recent experiences to aid with recall.

There was a lack of consistency among conceptualisations of acceptability and associated measures in WASH intervention studies, limiting their comparability and usefulness to inform intervention sustainability. While all 36 included studies claimed to measure acceptability, most did not provide a theoretical basis for their acceptability study design methods, and only four explicitly defined their acceptability measure [21–24]. The first limitation arising from such inconsistency is that studies that used subjective measures, such as ‘feelings towards’ the intervention, missed other potentially key components such as ‘willingness to purchase’ or levels of use and vice versa. The second limitation is that variation in definition limits the comparability of acceptability assessments of the same intervention in different populations.

A consistent approach to acceptability measurement in WASH is needed. To achieve this, we advocate the use of a theory-based a priori definition for acceptability research in WASH to inform measure development and assessment. An example of this is Sekhon et al.’s definition for acceptability, “a multi-faceted construct that reflects the extent to which people delivering or receiving a healthcare intervention consider it to be appropriate, based on anticipated or experienced cognitive and emotional responses to the intervention” [15]. Sekhon et al.’s theoretical framework of acceptability outlines seven component constructs that capture: attitude towards the intervention (affective attitude), burden, perceived effectiveness, ethicality, understanding (intervention coherence), opportunity costs, and participant’s belief in whether they can use it (self-efficacy). This theory-based definition outlines clear parameters of what acceptability is, while still allowing for varied forms of measurement based on the requirements of the research. Future research may include a greater examination of how adaptable the theoretical framework is in relation to WASH in different settings.

Some authors used validated behavioural frameworks, such as the health belief model (HBM) and the Integrated Behavioural Model for Water, Sanitation, and Hygiene (IBM-WASH), to design their acceptability measures [35,36,44]. IBM-WASH is a tool designed to identify and address individual and contextual factors that affect behavioural outcomes for WASH interventions [25]. IBM-WASH and similar frameworks designed for health promotion more broadly can be used contextualize or interpret acceptability findings.

This review should be interpreted in light of some limitations. First, the number of studies included in this review may have been limited by the search and inclusion criteria; where only articles that included the word “accept*” in the title, abstract or methods were searched for and retained in the final review. The implication of this is that WASH studies that measured related but not equivalent constructs, such as satisfaction, may have not appeared in the search and were not included. However, the aim of this review was to identify how "acceptability” was measured in WASH interventions. As such, the inclusion of studies that did not focus on acceptability would expand beyond the scope of this review and risk misinterpretation of the findings.

Second, this review was also limited to acceptability evaluations of WASH interventions published in English. This may represent a particular limitation for LMIC studies as researchers may prefer to publish in their own language for dissemination of study findings. Third, formative research, which informs the content and delivery of interventions, may involve acceptability components. It is possible that in restricting our review to specific interventions we have underestimated the number of prospective acceptability studies. However, this would not affect the number of studies reporting retrospective acceptability as a measure. The diversity of acceptability measurements and inclusion of a broad suite of interventions limited the potential for meta-analysis, however, a strength of this study was the breadth of WASH captured. While we found a degree of bias in the majority of research, we retained all studies that met the inclusion criteria because the exclusion of all low-quality studies would have considerably limited our review. As our focus is on acceptability measures, which include objective and subjective measures, rather than quantifiable outcomes our findings may be less impacted by the quality of the studies.

## Conclusions

We have identified four main areas that should be addressed for acceptability assessments in WASH. First, there is a need for acceptability evaluations in schools and healthcare facilities. Second, few studies conduct prospective evaluations which are useful for encouraging community collaboration, refining intervention design, and increasing initial uptake. Third, retrospective evaluations may contribute to sustained use, but their timing must be carefully considered. Finally, a clear and consistent approach to definition and measurement of acceptability in WASH development is needed. Inclusion of acceptability in the complex intervention development and evaluation cycle can contribute to improved effectiveness, sustainability and ultimately, use of resources to meet global development goals.

## Supporting information

S1 Table Inclusion and exclusion criteria. (DOCX)

S2 Table Additional extraction information. A file containing supplementary data tables including: Table A. For research articles published January 1990- December 2021 where the acceptability of water, sanitation and hygiene intervention was measured, by year. Table B. For research articles published January 1990- December 2021 where the acceptability of water, sanitation and hygiene intervention was measured, by year.
(DOCX)

S3 Table Risk of Bias and quality assessments. A file containing supplementary data tables including: Table A. Newcastle-Ottawa Quality Assessment (adapted from cross-sectional NoS). Table B. Cochrane assessment, measured based on intention-to-treat based on standard practice for reviews.
(DOCX)

S4 Table Acceptability measure categorisation. (DOCX)
