# Supplementary material for: Acceptability measures of water, sanitation and hygiene interventions in low- and middle-income countries, a systematic review
Source: PLoS Negl Trop Dis. 2022 Sep 12;16(9):e0010702. doi: 10.1371/journal.pntd.0010702 (PMC9499221; doi:10.1371/journal.pntd.0010702)
Supplement: S1 Table — (DOCX) [file pntd.0010702.s001.docx]

**S1 Table**

**Table A. Inclusion and exclusion criteria**

| **Research Objective** | To assess measures of acceptability of WASH (water, sanitation, hygiene) interventions in low and middle income countries.  Low- and middle income countries: those included on the Development Assistance Committee (DAC) - Organisation for Economic Co-operation and Development (OECD) list of official development assistance (OAD) 2021 by World Bank gross national income per capita[1]. |
| --- | --- |
| **Definition of acceptability** | To meet the aims of this review the search will not include synonyms of acceptability (e.g. satisfaction, approval, adherence) but will search on “accept*” only. |
| **Inclusion criteria** | 1. RCTs, quasi-experimental, qualitative studies 2. Intervention study (either a package of WASH interventions or a single water, sanitation or hygiene intervention 3. Mentions “accept” in title, abstract or methods 4. All languages 5. Published January 1990 -December 2021 |
| **Exclusion criteria** | 1. Conducted in a developed country (one not present on OEDC DAC List of ODA recipients) 2. Non-WASH intervention (dental, menstrual, sleep, clinical, chronic health outcome) or no intervention (formative research only) 3. Water quality assessment 4. Non-human study (plant or animal) 5. No mention of “Accept” in title, abstract or methods 6. Secondary research (review) 7. Conference proceedings, abstracts or workshop notes 8. Studies published before 1990 |
| **Search terms** | By Topic (Web of Science, Cochrane) or Title/Abstract (PubMed, Scopus)   1. WASH OR ‘water, sanitation and hygiene’ OR water OR sanitation OR hygiene OR toilet OR latrine OR handwash* AND 2. Intervention AND 3. Accept* |
| **Databases** | PubMed, Web of Science, SCOPUS, Cochrane Collaboration |

**References**

1. OECD. DAC List of ODA Recipients. 2021. Available from: https://www.oecd.org/dac/financing-sustainable-development/development-finance-standards/daclist.htm. [Accessed 14 Dec 2021]
