# Supplementary material for: Acceptability measures of water, sanitation and hygiene interventions in low- and middle-income countries, a systematic review
Source: PLoS Negl Trop Dis. 2022 Sep 12;16(9):e0010702. doi: 10.1371/journal.pntd.0010702 (PMC9499221; doi:10.1371/journal.pntd.0010702)
Supplement: S2 Table — A file containing supplementary data tables including: Table A. For research articles published January 1990- December 2021 where the acceptability of water, sanitation and hygiene intervention was measured, by year. Table B. For research articles published January 1990- December 2021 where the acceptability of water, sanitation and hygiene intervention was measured, by year. (DOCX) [file pntd.0010702.s002.docx]

**S2 Table: Additional extraction information**

**Table A. Additional extraction information– non-randomized designs.** For research articles published January 1990- December 2021 where the acceptability of water, sanitation and hygiene intervention was measured, by year**.**

| **Authors** | **Year** | **Title** | **Primary outcome** | **Use of assessment** | **Subsequent trials** |
| --- | --- | --- | --- | --- | --- |
| Aikhomu et al. [1] | 2000 | Acceptance and use of communal filtration units in guinea worm eradication | Adherence | Made recommendations - further use |  |
| Rainey et al. [2] | 2005 | Acceptability of solar disinfection of drinking water treatment in Kathmandu Valley, Nepal | Acceptability | Made recommendations - communication and implementation |  |
| Simms et al. [3] | 2005 | Sustainability and acceptability of latrine provision in The Gambia | Acceptability | Made recommendations - further use, communication and implementation |  |
| Rose et al. [4] | 2006 | Solar disinfection of water for diarrhoeal prevention in southern India | Diarrhoea (incidence, duration, severity) | Made recommendations - further use |  |
| Diallo et al. [5] | 2007 | Household latrine use, maintenance and acceptability in rural Zinder, Niger | Adherence | Made recommendations- further use, communication and implementation |  |
| Hulland et al. [6] | 2013 | Designing a handwashing station for infrastructure-restricted communities in Bangladesh using the integrated behavioural model for water, sanitation and hygiene interventions (IBM-WASH). | Acceptability | Made changes - design | Yes - 2 subsequent RCTs [7] |
| Francis et al. [8] | 2015 | Perception of drinking water safety and factors influencing acceptance and sustainability of a water quality intervention in rural southern India | Acceptability | Made recommendations- further use, communication and implementation |  |
| Hogarh et al. [9] | 2015 | Biosand filter as a household water treatment technology in Ghana and its ecobusiness potential: An assessment using a lifecycle approach | Ecobusiness potential | Made recommendations- further use, communication and implementation |  |
| Kundu et al. [10] | 2016 | Understanding social acceptability of arsenic-safe technologies in rural Bangladesh: a user-oriented analysis | Acceptability | Made recommendations- communication and implementation |  |
| Ashraf et al. [7] | 2017 | Nonrandomized Trial of Feasibility and Acceptability of Strategies for Promotion of Soapy Water as a Handwashing Agent in Rural Bangladesh | Adherence, uptake | Made changes - design |  |
| Hussain et al. [11] | 2017 | Assessment of the Acceptability and Feasibility of Child Potties for Safe Child Feces Disposal in Rural Bangladesh | Acceptability | Made recommendations- communication and implementation |  |
| Yeasmin et al. [12] | 2017 | Piloting a low-cost hardware intervention to reduce improper disposal of solid waste in communal toilets in low-income settlements in Dhaka, Bangladesh | Acceptability | Made changes - design, communication and implementation, Made recommendations- further use |  |
| Crider et al. [13] | 2018 | Can you taste it? Taste detection and acceptability thresholds for chlorine residual in drinking water in Dhaka, Bangladesh | Detection thresholds | Made recommendations- implementation |  |
| Sultana et al. [14] | 2018 | Acceptability and Feasibility of Sharing a Soapy Water System for Handwashing in a Low-Income Urban Community in Dhaka, Bangladesh: A Qualitative Study | Acceptability | Made recommendations, further use, design changes |  |
| Yeasmin et al. [15] | 2019 | Piloting a Shared Source Water Treatment Intervention among Elementary Schools in Bangladesh | Uptake | Made changes - design, communication and implementation, Made recommendations- further use | Yes, within study |
| Alam et al. [16] | 2020 | Barriers and Enabling Factors for Central and Household Level Water Treatment in a Refugee Setting: A Mixed-Method Study among Rohingyas in Cox's Bazar, Bangladesh | Barriers/Enablers | Made recommendations- communication and implementation |  |
| Bitew et al. [17] | 2020 | Barriers and Enabling Factors Associated with the Implementation of Household Solar Water Disinfection: A Qualitative Study in Northwest Ethiopia | Barriers/Enablers | Made recommendations- further use, communication and implementation |  |
| Campbell et al. [18] | 2020 | Facilitators and barriers to a family empowerment strategy to improve healthcare worker hand hygiene in a resource-limited setting | Uptake | Made changes - design, communication and implementation, Made recommendations- further use | Yes, within study |
| Guo et al. [19] | 2021 | Poor awareness and attitudes to sanitation servicing can impede China's Rural Toilet Revolution: Evidence from Western China | Compliance, Adherence | Made recommendations- communication and implementation |  |
| Sutherland et al. [20] | 2021 | Innovation for improved hand hygiene: Field testing the Autarky handwashing station in collaboration with informal settlement residents in Durban, South Africa | Acceptability | Made recommendations- further use, Made changes - communication and implementation |  |
| Thorseth et al. [21] | 2021 | An exploratory pilot study of the effect of modified hygiene kits on handwashing with soap among internally displaced persons in Ethiopia | Adherence | Made recommendations- design, communication and implementation |  |
| Yeasmin et al. [15] | 2021 | Could Alcohol-Based Hand Sanitizer Be an Option for Hand Hygiene for Households in Rural Bangladesh? | Adherence | Made changes - communication and implementation, between study phases | Yes, larger RCT |

**Table B. Additional extraction information – randomized designs.** For research articles published January 1990- December 2021 where the acceptability of water, sanitation and hygiene intervention was evaluated, by year.

| **Authors** | **Year** | **Title** | **Primary outcome** | **Use of assessment** | **Subsequent trials** |
| --- | --- | --- | --- | --- | --- |
| Firth et al. [22] | 2010 | Point-of-use interventions to decrease contamination of drinking water: a randomized, controlled pilot study on efficacy, effectiveness, and acceptability of closed containers, Moringa oleifera, and in-home chlorination in rural South India | Awareness, water contamination from sampling | Made recommendations- further use, communication and implementation |  |
| McGuigan et al. [23] | 2011 | High Compliance Randomized Controlled Field Trial of Solar Disinfection of Drinking Water and Its Impact on Childhood Diarrhea in Rural Cambodia | Diarrhoea incidence | Made recommendations- further use |  |
| Habib et al. [24] | 2013 | A study to evaluate the acceptability, feasibility and impact of packaged interventions ("Diarrhea Pack") for prevention and treatment of childhood diarrhea in rural Pakistan | Diarrhoea prevalence | Made recommendations- further use, communication and implementation |  |
| Rajaraman et al. [25] | 2014 | Implementing effective hygiene promotion: lessons from the process evaluation of an intervention to promote handwashing with soap in rural India | Process Evaluation | Made recommendations- further use |  |
| Biswas et al. [26] | 2017 | Provision versus promotion to develop a handwashing station: the effect on desired handwashing behaviour | Adherence | Made recommendations- further use, communication and implementation |  |
| Biran et al. [27] | 2018 | A Cluster-Randomized Trial to Evaluate the Impact of an Inclusive, Community-Led Total Sanitation Intervention on Sanitation Access for People with Disabilities in Malawi | Proportion of households that made changes to their latrines | Made recommendations- communication and implementation |  |
| Ditai et al. [28] | 2018 | Preventing neonatal sepsis in rural Uganda: a cross-over study comparing the tolerance and acceptability of three alcohol-based hand rub formulations | Rate of positive infection screen, use, mortality, quality of life, adverse events, contamination risk, | Made changes - design, communication and implementation, Made recommendations- further use | Yes, a cluster RCT [28] |
| Stone et al. [29] | 2018 | Educational intervention to reduce disease related to sub-optimal basic hygiene in Rwanda: initial evaluation and feasibility study | Diarrhoea | Made recommendations- further use | Yes - Training on DVDs initiated |
| Harrison et al. [30] | 2019 | We have to clean ourselves to ensure that our children are healthy and beautiful: findings from a qualitative assessment of a hand hygiene poster in rural Uganda | Compliance, Adherence | Made changes - design | Yes - Poster adapted to sticker and put on ABHR bottles |
| Rajasingham et al. [31] | 2019 | Evaluation of an Emergency Bulk Chlorination Project Targeting Drinking Water Vendors in Cholera-Affected Wards of Dar es Salaam and Morogoro, Tanzania | Adherence | Made recommendations- further use |  |
| Heitzinger et al. [32] | 2020 | Assessment of the Feasibility and Acceptability of Using Water Pasteurization Indicators to Increase Access to Safe Drinking Water in the Peruvian Amazon | Acceptability | Made changes - design | Yes - subsequent RCT |
| McGuiness et al. [33] | 2020 | Barriers and Enablers to Intervention Uptake and Health Reporting in a Water Intervention Trial in Rural India: A Qualitative Explanatory Study | Uptake | Explain quantitative, make recommendations | Yes - Post an RCT [34] |
| Ngasala et al. [35] | 2020 | Implementation of point-of-use water treatment methods in a rural Tanzanian community: A case study | Water quality | Made recommendations- further use, communication and implementation |  |
| Budge et al. [36] | 2021 | Multi-Sectoral Participatory Design of a BabyWASH Playspace for Rural Ethiopian Households | Acceptability | Made changes - design, communication and implementation, Made recommendations- further use | Yes, subsequent RCT 2021[37] |

**References**

1. Aikhomu SE, Brieger WR, Kale OO. Acceptance and use of communal filtration units in guinea worm eradication. Tropical Medicine and International Health. 2000;5(1):47-52.

2. Rainey RC, Harding AK. Acceptability of solar disinfection of drinking water treatment in Kathmandu Valley, Nepal. International Journal of Environmental Health Research. 2005;15(5):361-72.

3. Simms VM, Makalo P, Bailey RL, Emerson PM. Sustainability and acceptability of latrine provision in The Gambia. Transactions of the Royal Society of Tropical Medicine and Hygiene. 2005;99(8):631-7.

4. Rose A, Roy S, Abraham V, Holmgren G, George K, Balraj V, et al. Solar disinfection of water for diarrhoeal prevention in southern India. Archives of Disease in Childhood. 2006;91(2):139-41.

5. Diallo MO, Hopkins DR, Kane MS, Niandou S, Amadou A, Kadri B, et al. Household latrine use, maintenance and acceptability in rural Zinder, Niger. International Journal of Environmental Health Research. 2007;17(6):443-52.

6. Hulland KRS, Leontsini E, Dreibelbis R, Unicomb L, Afroz A, Dutta NC, et al. Designing a handwashing station for infrastructure-restricted communities in Bangladesh using the integrated behavioural model for water, sanitation and hygiene interventions (IBM-WASH). Bmc Public Health. 2013;13.

7. Ashraf S, Nizame FA, Islam M, Dutta NC, Yeasmin D, Akhter S, et al. Nonrandomized Trial of Feasibility and Acceptability of Strategies for Promotion of Soapy Water as a Handwashing Agent in Rural Bangladesh. American Journal of Tropical Medicine and Hygiene. 2017;96(2):421-9.

8. Francis MR, Nagarajan G, Sarkar R, Mohan VR, Kang G, Balraj V. Perception of drinking water safety and factors influencing acceptance and sustainability of a water quality intervention in rural southern India. BMC Public Health. 2015;15(1).

9. Hogarh JN, Sowunmi FA, Oluwafemi AP, Antwi-Agyei P, Nukpezah D, Atewamba CT. Biosand filter as a household water treatment technology in ghana and its ecobusiness potential: An assessment using a lifecycle approach. Journal of Environmental Accounting and Management. 2015;3(4):343-53.

10. Kundu DK, Gupta A, Mol APJ, Nasreen M. Understanding social acceptability of arsenic-safe technologies in rural Bangladesh: a user-oriented analysis. Water Policy. 2016;18(2):318-34.

11. Hussain F, Luby SP, Unicomb L, Leontsini E, Naushin T, Buckland AJ, et al. Assessment of the Acceptability and Feasibility of Child Potties for Safe Child Feces Disposal in Rural Bangladesh. American Journal of Tropical Medicine and Hygiene. 2017;97(2):469-76.

12. Yeasmin F, Luby SP, Saxton RE, Nizame FA, Alam M-U, Dutta NC, et al. Piloting a low-cost hardware intervention to reduce improper disposal of solid waste in communal toilets in low-income settlements in Dhaka, Bangladesh. BMC Public Health. 2017;17(1):682.

13. Crider Y, Sultana S, Unicomb L, Davis J, Luby SP, Pickering AJ. Can you taste it? Taste detection and acceptability thresholds for chlorine residual in drinking water in Dhaka, Bangladesh. Science of the Total Environment. 2018;613:840-6.

14. Sultana F, Unicomb LE, Nizame FA, Dutta NC, Ram PK, Luby SP, et al. Acceptability and Feasibility of Sharing a Soapy Water System for Handwashing in a Low-Income Urban Community in Dhaka, Bangladesh: A Qualitative Study. American Journal of Tropical Medicine and Hygiene. 2018;99(2):502-12.

15. Yeasmin F, Sultana F, Unicomb L, Nizame FA, Rahman M, Kabir H, et al. Piloting a Shared Source Water Treatment Intervention among Elementary Schools in Bangladesh. American Journal of Tropical Medicine and Hygiene. 2019;101(5):984-93.

16. Alam MU, Unicomb L, Ahasan SMM, Amin N, Biswas D, Ferdous S, et al. Barriers and Enabling Factors for Central and Household Level Water Treatment in a Refugee Setting: A Mixed-Method Study among Rohingyas in Cox's Bazar, Bangladesh. Water. 2020;12(11).

17. Bitew BD, Gete YK, Biks GA, Adafrie TT. Barriers and Enabling Factors Associated with the Implementation of Household Solar Water Disinfection: A Qualitative Study in Northwest Ethiopia. American Journal of Tropical Medicine and Hygiene. 2020;102(2):458-67.

18. Campbell JI, Pham TT, Le T, Dang TTH, Chandonnet CJ, Truong TH, et al. Facilitators and barriers to a family empowerment strategy to improve healthcare worker hand hygiene in a resource-limited setting. Am J Infect Control. 2020;48(12):1485-90.

19. Guo S, Zhou X, Simha P, Mercado LFP, Lv Y, Li Z. Poor awareness and attitudes to sanitation servicing can impede China's Rural Toilet Revolution: Evidence from Western China. Science of The Total Environment. 2021;794:148660.

20. Sutherland C, Reynaert E, Sindall RC, Riechmann ME, Magwaza F, Lienert J, et al. Innovation for improved hand hygiene: Field testing the Autarky handwashing station in collaboration with informal settlement residents in Durban, South Africa. Science of the Total Environment. 2021;796.

21. Thorseth AH, Heath T, Sisay A, Hamo M, White S. An exploratory pilot study of the effect of modified hygiene kits on handwashing with soap among internally displaced persons in Ethiopia. Conflict and Health. 2021;15(1).

22. Firth J, Balraj V, Muliyil J, Roy S, Rani LM, Chandresekhar R, et al. Point-of-use interventions to decrease contamination of drinking water: a randomized, controlled pilot study on efficacy, effectiveness, and acceptability of closed containers, Moringa oleifera, and in-home chlorination in rural South India. The American journal of tropical medicine and hygiene. 2010;82(5):759-65.

23. McGuigan KG, Samaiyar P, du Preez M, Conroy RM. High Compliance Randomized Controlled Field Trial of Solar Disinfection of Drinking Water and Its Impact on Childhood Diarrhea in Rural Cambodia. Environmental Science & Technology. 2011;45(18):7862-7.

24. Habib MA, Soofi S, Sadiq K, Samejo T, Hussain M, Mirani M, et al. A study to evaluate the acceptability, feasibility and impact of packaged interventions ("Diarrhea Pack") for prevention and treatment of childhood diarrhea in rural Pakistan. Bmc Public Health. 2013;13.

25. Rajaraman D, Varadharajan KS, Greenland K, Curtis V, Kumar R, Schmidt WP, et al. Implementing effective hygiene promotion: lessons from the process evaluation of an intervention to promote handwashing with soap in rural India. Bmc Public Health. 2014;14.

26. Biswas D, Nizame FA, Sanghvi T, Roy S, Luby SP, Unicomb LE. Provision versus promotion to develop a handwashing station: the effect on desired handwashing behavior. Bmc Public Health. 2017;17.

27. Biran A, Danquah L, Chunga J, Schmidt WP, Holm R, Itimu-Phiri A, et al. A Cluster-Randomized Trial to Evaluate the Impact of an Inclusive, Community-Led Total Sanitation Intervention on Sanitation Access for People with Disabilities in Malawi. American Journal of Tropical Medicine and Hygiene. 2018;98(4):984-94.

28. Ditai J, Mudoola M, Gladstone M, Abeso J, Dusabe-Richards J, Adengo M, et al. Preventing neonatal sepsis in rural Uganda: a cross-over study comparing the tolerance and acceptability of three alcohol-based hand rub formulations. BMC Public Health. 2018;18(1):1279.

29. Stone MA, Ndagijimana H. Educational intervention to reduce disease related to sub-optimal basic hygiene in Rwanda: initial evaluation and feasibility study. Pilot Feasibility Stud. 2018;4:4.

30. Harrison BL, Ogara C, Gladstone M, Carrol ED, Dusabe-Richards J, Medina-Lara A, et al. "We have to clean ourselves to ensure that our children are healthy and beautiful": findings from a qualitative assessment of a hand hygiene poster in rural Uganda. Bmc Public Health. 2019;19.

31. Rajasingham A, Hardy C, Kamwaga S, Sebunya K, Massa K, Mulungu J, et al. Evaluation of an Emergency Bulk Chlorination Project Targeting Drinking Water Vendors in Cholera-Affected Wards of Dar es Salaam and Morogoro, Tanzania. American Journal of Tropical Medicine and Hygiene. 2019;100(6):1335-41.

32. Heitzinger K, Hawes SE, Rocha CA, Alvarez C, Evans CA. Assessment of the Feasibility and Acceptability of Using Water Pasteurization Indicators to Increase Access to Safe Drinking Water in the Peruvian Amazon. American Journal of Tropical Medicine and Hygiene. 2020;103(1):455-64.

33. McGuinness SL, O'Toole J, Giriyan A, Gaonkar CA, Reddy V, Patil K, et al. Perceptions, experiences and acceptability of a water intervention using riverbank filtration technology in rural India. American journal of tropical medicine and hygiene. 2018;99(4):431‐.

34. McGuinness SL, O’Toole J, Forbes AB, Boving TB, Patil K, D’Souza F, et al. A Stepped Wedge Cluster-Randomized Trial Assessing the Impact of a Riverbank Filtration Intervention to Improve Access to Safe Water on Health in Rural India. The American Journal of Tropical Medicine and Hygiene. 2020;102(3):497-506.

35. Ngasala TM, Masten SJ, Cohen C, Ravitz D, Mwita EJ. Implementation of point-of-use water treatment methods in a rural tanzanian community: A case study. J Water Sanit Hyg De. 2020;10(4):1012-8.

36. Budge S, Parker A, Hutchings P, Garbutt C, Rosenbaum J, Tulu T, et al. Multi-Sectoral Participatory Design of a BabyWASH Playspace for Rural Ethiopian Households. American Journal of Tropical Medicine and Hygiene. 2021;104(3):884-97.

37. Budge S, Hutchings P, Parker A, Tyrrel S, Norton S, Garbutt C, et al. A randomised controlled feasibility trial of a BabyWASH household playspace: The CAMPI study. PLOS Neglected Tropical Diseases. 2021;15(7):e0009514.

**List of Legends**

**Table A.** For research articles published January 1990- December 2021 where the acceptability of water, sanitation and hygiene intervention was measured, by year**.**

**Table B.** For research articles published January 1990- December 2021 where the acceptability of water, sanitation and hygiene intervention was measured, by year**.**
