# Supplementary material for: Acceptability measures of water, sanitation and hygiene interventions in low- and middle-income countries, a systematic review
Source: PLoS Negl Trop Dis. 2022 Sep 12;16(9):e0010702. doi: 10.1371/journal.pntd.0010702 (PMC9499221; doi:10.1371/journal.pntd.0010702)
Supplement: S3 Table — A file containing supplementary data tables including: Table A. Newcastle-Ottawa Quality Assessment (adapted from cross-sectional NoS). Table B. Cochrane assessment, measured based on intention-to-treat based on standard practice for reviews. (DOCX) [file pntd.0010702.s003.docx]

**S3 Table: Risk of Bias and Quality Assessments**

**Table A. Risk of Bias assessment of cross-sectional studies.** Newcastle-Ottawa Quality Assessment (adapted from cross-sectional NoS)[1]

| Authors | Selection | | | | Comparability | Outcome | | Score | Quality |
| --- | --- | --- | --- | --- | --- | --- | --- | --- | --- |
|  | **Representativeness of sample** | **Sample size** | **Respondents** | **Ascertainment of exposure** | **Control of relevant factors to study** | **Assessment** | **Statistical analyses** | **/10** |  |
| Aikomu et al. (2000) [2] | * |  |  | * | * | * |  | 4 | Moderate |
| Rainey et al. (2005) [3] | * |  |  | ** | * | ** |  | 6 | Moderate |
| Simms et al. (2005) [4] | * |  |  | * | * | ** |  | 5 | Moderate |
| Rose et al. (2006) [5] | * |  |  | * | * | ** | * | 6 | Moderate |
| Diallo et al. (2007) [6] | * |  | * | * | * | ** |  | 6 | Moderate |
| Hulland et al. (2013) [7] | * |  |  | ** | ** | ** |  | 7 | High |
| Francis et al. (2015) [8] | * |  |  | * | * | * |  | 4 | Moderate |
| Hogarh et al. (2015) [9] | * |  |  | * | * | * | * | 5 | Moderate |
| Kundu et al. (2016) [10] | * |  |  | * | ** | * |  | 5 | Moderate |
| Ashraf et al. (2017) [11] | * | * |  | ** | * | ** | * | 8 | High |
| Hussain et al. (2017) [12] | * |  |  | ** | * | ** |  | 6 | Moderate |
| Yeasmin et al. (2017) [13] |  |  |  | ** | * | ** |  | 5 | Moderate |
| Crider et al. (2018) [14] | * | * |  | * | * | ** | * | 7 | High |
| Ditai et al. (2018) [15] | * | * | * | ** | * | * |  | 7 | High |
| Sultana et al. (2018) [16] | * |  |  | ** | ** | * |  | 6 | Moderate |
| Rajasingham et al. (2019) [17] | * |  |  | * | * | ** | * | 6 | Moderate |
| Yeasmin et al. (2019) [18] | * |  |  | ** | * | ** |  | 6 | Moderate |
| Alam et al. (2020) [19] |  |  |  | * | ** | ** |  | 5 | Moderate |
| Bitew et al. (2020) [20] | * |  |  | * | * | * |  | 4 | Moderate |
| Campbell et al. (2020) [21] | * |  |  | * | * | * |  | 4 | Moderate |
| Guo et al. (2021) [22] | * |  |  | * | ** | ** |  | 6 | Moderate |
| Sutherland et al. (2021) [23] | * | * |  | * | * | * |  | 5 | Moderate |
| Thorseth et al. (2021) [24] | * | * |  | * | * | ** | * | 7 | High |
| Yeasmin et al. (2021) [25] | * |  |  | ** | * | ** |  | 6 | Moderate |

**Table B. Risk of Bias Assessment: randomized control trials.** Cochrane assessment, measured based on intention-to-treat based on standard practice for reviews[26]

| Authors | 1. Randomisation | 2. Assignment to intervention | 3. Incomplete outcome data | 4. Selective outcome measurement | 5. Selective outcome reporting | Overall Score |
| --- | --- | --- | --- | --- | --- | --- |
| Firth et al. (2010) [27] | Low risk | Low risk | Low risk | Some concerns | High risk | High risk |
| McGuigan et al. (2011) [28] | Low risk | Low risk | Low risk | Low risk | Some concerns | Some concerns |
| Heitzinger et al. (2020) [29] | Low risk | Low risk | Low risk | Some concerns | Some concerns | Some concerns |
| McGuinness et al. (2018) [30] | Low risk | Low risk | Low risk | Low risk | Some concerns | Some concerns |
| Ngasala et al. (2020) [31] | Low risk | Low risk | Low risk | Low risk | Some concerns | Some concerns |
| Budge et al. (2021) [32] | Low risk | High risk | Low risk | High risk | Low risk | High risk |

**Table C. Risk of Bias Assessment: cluster-randomized control trials.** Cochrane assessment, measured based on intention-to-treat based on standard practice for reviews^2^

| Authors | 1a. Randomisation | 1b. Recruitment | 2. Assignment to intervention | 3. Incomplete outcome data | 4. Selective outcome measurement | 5. Selective outcome reporting | Overall Score |
| --- | --- | --- | --- | --- | --- | --- | --- |
| Habib et al. (2013) [33] | Low risk | Low risk | Low risk | High risk | Low risk | High risk | High risk |
| Rajaraman et al. (2014) [34] | Low risk | Low risk | Some concerns | Low risk | Low risk | Some concerns | Some concerns |
| Biswas et al. (2017) [35] | Low risk | Low risk | Low risk | Low risk | Low risk | Some concerns | Some concerns |
| Biran et al. (2018) [36] | Low risk | Low risk | Some concerns | Low risk | High risk | High risk | High risk |
| Stone et al. (2018) [37] | Some concerns | Low risk | Low risk | High risk | Low risk | Some concerns | High risk |
| Harrison et al. (2019) [38] | Some concerns | High risk | Low risk | Low risk | High risk | Some concerns | High risk |

**References**

1. Wells G, Shea B, O’Connell D, Peterson J, Welch V, Losos M, et al. The Newcastle-Ottawa Scale (NOS) for assessing the quality of nonrandomised studies in meta-analyses. Oxford; 2000. Available from: http://www.ohri.ca/programs/clinical_epidemiology/oxford.asp.

2. Aikhomu SE, Brieger WR, Kale OO. Acceptance and use of communal filtration units in guinea worm eradication. Tropical Medicine and International Health. 2000;5(1):47-52.

3. Rainey RC, Harding AK. Acceptability of solar disinfection of drinking water treatment in Kathmandu Valley, Nepal. International Journal of Environmental Health Research. 2005;15(5):361-72.

4. Simms VM, Makalo P, Bailey RL, Emerson PM. Sustainability and acceptability of latrine provision in The Gambia. Transactions of the Royal Society of Tropical Medicine and Hygiene. 2005;99(8):631-7.

5. Rose A, Roy S, Abraham V, Holmgren G, George K, Balraj V, et al. Solar disinfection of water for diarrhoeal prevention in southern India. Archives of Disease in Childhood. 2006;91(2):139-41.

6. Diallo MO, Hopkins DR, Kane MS, Niandou S, Amadou A, Kadri B, et al. Household latrine use, maintenance and acceptability in rural Zinder, Niger. International Journal of Environmental Health Research. 2007;17(6):443-52.

7. Hulland KRS, Leontsini E, Dreibelbis R, Unicomb L, Afroz A, Dutta NC, et al. Designing a handwashing station for infrastructure-restricted communities in Bangladesh using the integrated behavioural model for water, sanitation and hygiene interventions (IBM-WASH). Bmc Public Health. 2013;13.

8. Francis MR, Nagarajan G, Sarkar R, Mohan VR, Kang G, Balraj V. Perception of drinking water safety and factors influencing acceptance and sustainability of a water quality intervention in rural southern India. BMC Public Health. 2015;15(1).

9. Hogarh JN, Sowunmi FA, Oluwafemi AP, Antwi-Agyei P, Nukpezah D, Atewamba CT. Biosand filter as a household water treatment technology in ghana and its ecobusiness potential: An assessment using a lifecycle approach. Journal of Environmental Accounting and Management. 2015;3(4):343-53.

10. Kundu DK, Gupta A, Mol APJ, Nasreen M. Understanding social acceptability of arsenic-safe technologies in rural Bangladesh: a user-oriented analysis. Water Policy. 2016;18(2):318-34.

11. Ashraf S, Nizame FA, Islam M, Dutta NC, Yeasmin D, Akhter S, et al. Nonrandomized Trial of Feasibility and Acceptability of Strategies for Promotion of Soapy Water as a Handwashing Agent in Rural Bangladesh. American Journal of Tropical Medicine and Hygiene. 2017;96(2):421-9.

12. Hussain F, Luby SP, Unicomb L, Leontsini E, Naushin T, Buckland AJ, et al. Assessment of the Acceptability and Feasibility of Child Potties for Safe Child Feces Disposal in Rural Bangladesh. American Journal of Tropical Medicine and Hygiene. 2017;97(2):469-76.

13. Yeasmin F, Luby SP, Saxton RE, Nizame FA, Alam M-U, Dutta NC, et al. Piloting a low-cost hardware intervention to reduce improper disposal of solid waste in communal toilets in low-income settlements in Dhaka, Bangladesh. BMC Public Health. 2017;17(1):682.

14. Crider Y, Sultana S, Unicomb L, Davis J, Luby SP, Pickering AJ. Can you taste it? Taste detection and acceptability thresholds for chlorine residual in drinking water in Dhaka, Bangladesh. Science of the Total Environment. 2018;613:840-6.

15. Ditai J, Mudoola M, Gladstone M, Abeso J, Dusabe-Richards J, Adengo M, et al. Preventing neonatal sepsis in rural Uganda: a cross-over study comparing the tolerance and acceptability of three alcohol-based hand rub formulations. BMC Public Health. 2018;18(1):1279.

16. Sultana F, Unicomb LE, Nizame FA, Dutta NC, Ram PK, Luby SP, et al. Acceptability and Feasibility of Sharing a Soapy Water System for Handwashing in a Low-Income Urban Community in Dhaka, Bangladesh: A Qualitative Study. American Journal of Tropical Medicine and Hygiene. 2018;99(2):502-12.

17. Rajasingham A, Hardy C, Kamwaga S, Sebunya K, Massa K, Mulungu J, et al. Evaluation of an Emergency Bulk Chlorination Project Targeting Drinking Water Vendors in Cholera-Affected Wards of Dar es Salaam and Morogoro, Tanzania. American Journal of Tropical Medicine and Hygiene. 2019;100(6):1335-41.

18. Yeasmin F, Sultana F, Unicomb L, Nizame FA, Rahman M, Kabir H, et al. Piloting a Shared Source Water Treatment Intervention among Elementary Schools in Bangladesh. American Journal of Tropical Medicine and Hygiene. 2019;101(5):984-93.

19. Alam MU, Unicomb L, Ahasan SMM, Amin N, Biswas D, Ferdous S, et al. Barriers and Enabling Factors for Central and Household Level Water Treatment in a Refugee Setting: A Mixed-Method Study among Rohingyas in Cox's Bazar, Bangladesh. Water. 2020;12(11).

20. Bitew BD, Gete YK, Biks GA, Adafrie TT. Barriers and Enabling Factors Associated with the Implementation of Household Solar Water Disinfection: A Qualitative Study in Northwest Ethiopia. American Journal of Tropical Medicine and Hygiene. 2020;102(2):458-67.

21. Campbell JI, Pham TT, Le T, Dang TTH, Chandonnet CJ, Truong TH, et al. Facilitators and barriers to a family empowerment strategy to improve healthcare worker hand hygiene in a resource-limited setting. Am J Infect Control. 2020;48(12):1485-90.

22. Guo S, Zhou X, Simha P, Mercado LFP, Lv Y, Li Z. Poor awareness and attitudes to sanitation servicing can impede China's Rural Toilet Revolution: Evidence from Western China. Science of The Total Environment. 2021;794:148660.

23. Sutherland C, Reynaert E, Sindall RC, Riechmann ME, Magwaza F, Lienert J, et al. Innovation for improved hand hygiene: Field testing the Autarky handwashing station in collaboration with informal settlement residents in Durban, South Africa. Science of the Total Environment. 2021;796.

24. Thorseth AH, Heath T, Sisay A, Hamo M, White S. An exploratory pilot study of the effect of modified hygiene kits on handwashing with soap among internally displaced persons in Ethiopia. Conflict and Health. 2021;15(1).

25. Yeasmin D, Dutta NC, Nizame FA, Rahman MJ, Ashraf S, Ram PK, et al. Could Alcohol-Based Hand Sanitizer Be an Option for Hand Hygiene for Households in Rural Bangladesh? American Journal of Tropical Medicine and Hygiene. 2021;104(3):874-83.

26. Higgins JPT, Thomas J, Chandler J, Cumpston M, Li T, Page MJ, Welch VA (editors). Cochrane Handbook for Systematic Reviews of Interventions version 6.3 (updated February 2022). Cochrane, 2022. Available from www.training.cochrane.org/handbook.

27. Firth J, Balraj V, Muliyil J, Roy S, Rani LM, Chandresekhar R, et al. Point-of-use interventions to decrease contamination of drinking water: a randomized, controlled pilot study on efficacy, effectiveness, and acceptability of closed containers, Moringa oleifera, and in-home chlorination in rural South India. The American journal of tropical medicine and hygiene. 2010;82(5):759-65.

28. McGuigan KG, Samaiyar P, du Preez M, Conroy RM. High Compliance Randomized Controlled Field Trial of Solar Disinfection of Drinking Water and Its Impact on Childhood Diarrhea in Rural Cambodia. Environmental Science & Technology. 2011;45(18):7862-7.

29. Heitzinger K, Hawes SE, Rocha CA, Alvarez C, Evans CA. Assessment of the Feasibility and Acceptability of Using Water Pasteurization Indicators to Increase Access to Safe Drinking Water in the Peruvian Amazon. American Journal of Tropical Medicine and Hygiene. 2020;103(1):455-64.

30. McGuinness SL, O'Toole J, Giriyan A, Gaonkar CA, Reddy V, Patil K, et al. Perceptions, experiences and acceptability of a water intervention using riverbank filtration technology in rural India. American journal of tropical medicine and hygiene. 2018;99(4):431‐.

31. Ngasala TM, Masten SJ, Cohen C, Ravitz D, Mwita EJ. Implementation of point-of-use water treatment methods in a rural tanzanian community: A case study. J Water Sanit Hyg De. 2020;10(4):1012-8.

32. Budge S, Parker A, Hutchings P, Garbutt C, Rosenbaum J, Tulu T, et al. Multi-Sectoral Participatory Design of a BabyWASH Playspace for Rural Ethiopian Households. American Journal of Tropical Medicine and Hygiene. 2021;104(3):884-97.

33. Habib MA, Soofi S, Sadiq K, Samejo T, Hussain M, Mirani M, et al. A study to evaluate the acceptability, feasibility and impact of packaged interventions ("Diarrhea Pack") for prevention and treatment of childhood diarrhea in rural Pakistan. Bmc Public Health. 2013;13.

34. Rajaraman D, Varadharajan KS, Greenland K, Curtis V, Kumar R, Schmidt WP, et al. Implementing effective hygiene promotion: lessons from the process evaluation of an intervention to promote handwashing with soap in rural India. Bmc Public Health. 2014;14.

35. Biswas D, Nizame FA, Sanghvi T, Roy S, Luby SP, Unicomb LE. Provision versus promotion to develop a handwashing station: the effect on desired handwashing behavior. Bmc Public Health. 2017;17.

36. Biran A, Danquah L, Chunga J, Schmidt WP, Holm R, Itimu-Phiri A, et al. A Cluster-Randomized Trial to Evaluate the Impact of an Inclusive, Community-Led Total Sanitation Intervention on Sanitation Access for People with Disabilities in Malawi. American Journal of Tropical Medicine and Hygiene. 2018;98(4):984-94.

37. Stone MA, Ndagijimana H. Educational intervention to reduce disease related to sub-optimal basic hygiene in Rwanda: initial evaluation and feasibility study. Pilot Feasibility Stud. 2018;4:4.

38. Harrison BL, Ogara C, Gladstone M, Carrol ED, Dusabe-Richards J, Medina-Lara A, et al. "We have to clean ourselves to ensure that our children are healthy and beautiful": findings from a qualitative assessment of a hand hygiene poster in rural Uganda. Bmc Public Health. 2019;19.

**List of Legends**

Table A. Newcastle-Ottawa Quality Assessment (adapted from cross-sectional NoS) [1]

Table B. Cochrane assessment, measured based on intention-to-treat based on standard practice for reviews [26]
