# Supplementary material for: Acceptability measures of water, sanitation and hygiene interventions in low- and middle-income countries, a systematic review
Source: PLoS Negl Trop Dis. 2022 Sep 12;16(9):e0010702. doi: 10.1371/journal.pntd.0010702 (PMC9499221; doi:10.1371/journal.pntd.0010702)
Supplement: S4 Table — (DOCX) [file pntd.0010702.s004.docx]

**S4 Table: Acceptability measure categorisation**

**Table A. Definitions of acceptability in included studies.** The majority of our included WASH acceptability studies did not provide an explicit definition of acceptability. To facilitate comparisons between the included articles, we developed a categorisation system for the acceptability measures used across the 36 papers. Acceptability measures were grouped based on similar characteristics. The article definition categorisations were independently reviewed by two authors (RH, SYO). Definitions with quotations “ ” were supplied by the study authors, all others are implicit definitions.

| **Author** | **Acceptability definition** | **Category** |
| --- | --- | --- |
| Aikhomu et al. (2000) [1] | Perceptions of positive and negative features | Feelings |
| Budge et al. (2021) [2] | Acceptability of use, acceptability of design, and time use | Feelings |
| Heitzinger et al. (2020) [3] | “Participant’s satisfaction with use of the models” | Feelings |
| Ngasala et al. (2020) [4] | Attitude towards the intervention | Feelings |
| Simms et al. (2005) [5] | Satisfaction (happy/ unhappy) | Feelings |
| Crider et al. (2018) [6] | Perceived taste acceptability threshold | Feelings |
| Rajaraman et al. (2014) [7] | “Things liked and not liked” | Feelings |
| Ditai et al. (2018) [8] | Overall satisfaction | Feelings |
| Biswas et al. (2017) [9] | Motivations, ease of use, costs, barriers, “what they liked” | Feelings; motivations and barriers |
| Rose et al. (2006) [10] | Feelings towards the intervention, ease, cost, limitations | Feelings; motivations and barriers |
| Thorseth et al. (2021) [11] | Likes and dislikes, desirability, pleasantness, long lasting, familiarity, want, likeliness of use, effective, easy, water-saving | Feelings; motivations and barriers |
| Hulland et al. (2013) [12] | Appropriateness and satisfaction with handwashing station, agreement to install, maintain and use. | Feelings; Willingness |
| Hogarh et al. (2015) [13] | Willingness to purchase, interest | Feelings; Willingness |
| Ashraf et al. (2017) [14] | IBM-WASH framework: convenience/ease of use, perceived value and sharing, motivations for use, experiences, and barriers | Framework |
| McGuiness et al. (2020) [15] | COB-M framework: identification of barriers and enablers | Framework |
| Rainey et al. (2005) [16] | Based on Health Belief Model | Framework |
| Sultana et al. (2018) [17] | A separate construct to satisfaction, based on IBM-WASH framework | Framework |
| Yeasmin et al. (2017) [18] | IBM-WASH Framework: Perceptions, benefits and barriers | Framework |
| Yeasmin et al. (2021) [19] | IBM-WASH Framework: self-reported use rates as an indicator of acceptability | Framework |
| Alam et al. (2020) [20] | Barriers and motivations | Motivations and barriers |
| Diallo et al. (2017) [21] | Advantages and disadvantages of latrine use | Motivations and barriers |
| Campbell et al. (2020) [22] | No discernible definition | None |
| Yeasmin et al. (2019) [23] | No discernible definition | None |
| Bitew et al. (2020) [24] | Cultural acceptance: Barriers and enablers | Sociocultural values and norms |
| Guo et al. (2021) [25] | Perceived social acceptability | Sociocultural values and norms |
| Sutherland et al. (2021) [26] | Social acceptability, feelings towards site | Sociocultural values and norms; feelings |
| McGuigan et al. (2011) [27] | Use after 6 months taken to be culturally acceptable | Sociocultural values and norms; use |
| Kundu et al. (2016) [28] | "social acceptance i.e. the willingness of users to receive and use a technology" - They have a section "conceptualizing social acceptability: definition and factors" | Sociocultural values and norms; willingness |
| Harrison et al. (2019) [29] | Understanding, compliance to actions on poster, | Use; feelings |
| Firth et al. (2010) [30] | Satisfaction, interest, compliance, preference | Use; feelings |
| Rajasingham et al. (2019) [31] | Use of the water treatments, ease, perceptions of customer preferences | Use; feelings |
| Stone et al. (2018) [32] | Attendance at the health session, positivity towards activity | Use; feelings |
| Biran et al. (2018) [33] | Whether it was offensive, whether they were willing to do it | Willingness |
| Francis et al. (2015) [34] | Based on support for the intervention, willingness to pay for clean water | Willingness |
| Habib et al. (2013) [35] | Usage, perceived effectiveness, willingness to purchase | Willingness |
| Hussain et al. (2017) [36] | "An acceptable behaviour is one in which participants are willing to adopt and practice, that is feasible, practical, beneficial, and can be adjusted through negotiation" | Willingness; motivations and barriers |

**References**

1. Aikhomu SE, Brieger WR, Kale OO. Acceptance and use of communal filtration units in guinea worm eradication. Tropical Medicine and International Health. 2000;5(1):47-52.

2. Budge S, Parker A, Hutchings P, Garbutt C, Rosenbaum J, Tulu T, et al. Multi-Sectoral Participatory Design of a BabyWASH Playspace for Rural Ethiopian Households. American Journal of Tropical Medicine and Hygiene. 2021;104(3):884-97.

3. Heitzinger K, Hawes SE, Rocha CA, Alvarez C, Evans CA. Assessment of the Feasibility and Acceptability of Using Water Pasteurization Indicators to Increase Access to Safe Drinking Water in the Peruvian Amazon. American Journal of Tropical Medicine and Hygiene. 2020;103(1):455-64.

4. Ngasala TM, Masten SJ, Cohen C, Ravitz D, Mwita EJ. Implementation of point-of-use water treatment methods in a rural tanzanian community: A case study. J Water Sanit Hyg De. 2020;10(4):1012-8.

5. Simms VM, Makalo P, Bailey RL, Emerson PM. Sustainability and acceptability of latrine provision in The Gambia. Transactions of the Royal Society of Tropical Medicine and Hygiene. 2005;99(8):631-7.

6. Crider Y, Sultana S, Unicomb L, Davis J, Luby SP, Pickering AJ. Can you taste it? Taste detection and acceptability thresholds for chlorine residual in drinking water in Dhaka, Bangladesh. Science of the Total Environment. 2018;613:840-6.

7. Rajaraman D, Varadharajan KS, Greenland K, Curtis V, Kumar R, Schmidt WP, et al. Implementing effective hygiene promotion: lessons from the process evaluation of an intervention to promote handwashing with soap in rural India. Bmc Public Health. 2014;14.

8. Ditai J, Mudoola M, Gladstone M, Abeso J, Dusabe-Richards J, Adengo M, et al. Preventing neonatal sepsis in rural Uganda: a cross-over study comparing the tolerance and acceptability of three alcohol-based hand rub formulations. BMC Public Health. 2018;18(1):1279.

9. Biswas D, Nizame FA, Sanghvi T, Roy S, Luby SP, Unicomb LE. Provision versus promotion to develop a handwashing station: the effect on desired handwashing behavior. Bmc Public Health. 2017;17.

10. Rose A, Roy S, Abraham V, Holmgren G, George K, Balraj V, et al. Solar disinfection of water for diarrhoeal prevention in southern India. Archives of Disease in Childhood. 2006;91(2):139-41.

11. Thorseth AH, Heath T, Sisay A, Hamo M, White S. An exploratory pilot study of the effect of modified hygiene kits on handwashing with soap among internally displaced persons in Ethiopia. Conflict and Health. 2021;15(1).

12. Hulland KRS, Leontsini E, Dreibelbis R, Unicomb L, Afroz A, Dutta NC, et al. Designing a handwashing station for infrastructure-restricted communities in Bangladesh using the integrated behavioural model for water, sanitation and hygiene interventions (IBM-WASH). Bmc Public Health. 2013;13.

13. Hogarh JN, Sowunmi FA, Oluwafemi AP, Antwi-Agyei P, Nukpezah D, Atewamba CT. Biosand filter as a household water treatment technology in ghana and its ecobusiness potential: An assessment using a lifecycle approach. Journal of Environmental Accounting and Management. 2015;3(4):343-53.

14. Ashraf S, Nizame FA, Islam M, Dutta NC, Yeasmin D, Akhter S, et al. Nonrandomized Trial of Feasibility and Acceptability of Strategies for Promotion of Soapy Water as a Handwashing Agent in Rural Bangladesh. American Journal of Tropical Medicine and Hygiene. 2017;96(2):421-9.

15. McGuinness SL, O’Toole J, Forbes AB, Boving TB, Patil K, D’Souza F, et al. A Stepped Wedge Cluster-Randomized Trial Assessing the Impact of a Riverbank Filtration Intervention to Improve Access to Safe Water on Health in Rural India. The American Journal of Tropical Medicine and Hygiene. 2020;102(3):497-506.

16. Rainey RC, Harding AK. Acceptability of solar disinfection of drinking water treatment in Kathmandu Valley, Nepal. International Journal of Environmental Health Research. 2005;15(5):361-72.

17. Sultana F, Unicomb LE, Nizame FA, Dutta NC, Ram PK, Luby SP, et al. Acceptability and Feasibility of Sharing a Soapy Water System for Handwashing in a Low-Income Urban Community in Dhaka, Bangladesh: A Qualitative Study. American Journal of Tropical Medicine and Hygiene. 2018;99(2):502-12.

18. Yeasmin F, Luby SP, Saxton RE, Nizame FA, Alam M-U, Dutta NC, et al. Piloting a low-cost hardware intervention to reduce improper disposal of solid waste in communal toilets in low-income settlements in Dhaka, Bangladesh. BMC Public Health. 2017;17(1):682.

19. Yeasmin D, Dutta NC, Nizame FA, Rahman MJ, Ashraf S, Ram PK, et al. Could Alcohol-Based Hand Sanitizer Be an Option for Hand Hygiene for Households in Rural Bangladesh? American Journal of Tropical Medicine and Hygiene. 2021;104(3):874-83.

20. Alam MU, Unicomb L, Ahasan SMM, Amin N, Biswas D, Ferdous S, et al. Barriers and Enabling Factors for Central and Household Level Water Treatment in a Refugee Setting: A Mixed-Method Study among Rohingyas in Cox's Bazar, Bangladesh. Water. 2020;12(11).

21. Diallo MO, Hopkins DR, Kane MS, Niandou S, Amadou A, Kadri B, et al. Household latrine use, maintenance and acceptability in rural Zinder, Niger. International Journal of Environmental Health Research. 2007;17(6):443-52.

22. Campbell JI, Pham TT, Le T, Dang TTH, Chandonnet CJ, Truong TH, et al. Facilitators and barriers to a family empowerment strategy to improve healthcare worker hand hygiene in a resource-limited setting. Am J Infect Control. 2020;48(12):1485-90.

23. Yeasmin F, Sultana F, Unicomb L, Nizame FA, Rahman M, Kabir H, et al. Piloting a Shared Source Water Treatment Intervention among Elementary Schools in Bangladesh. American Journal of Tropical Medicine and Hygiene. 2019;101(5):984-93.

24. Bitew BD, Gete YK, Biks GA, Adafrie TT. Barriers and Enabling Factors Associated with the Implementation of Household Solar Water Disinfection: A Qualitative Study in Northwest Ethiopia. American Journal of Tropical Medicine and Hygiene. 2020;102(2):458-67.

25. Guo S, Zhou X, Simha P, Mercado LFP, Lv Y, Li Z. Poor awareness and attitudes to sanitation servicing can impede China's Rural Toilet Revolution: Evidence from Western China. Science of The Total Environment. 2021;794:148660.

26. Sutherland C, Reynaert E, Sindall RC, Riechmann ME, Magwaza F, Lienert J, et al. Innovation for improved hand hygiene: Field testing the Autarky handwashing station in collaboration with informal settlement residents in Durban, South Africa. Science of the Total Environment. 2021;796.

27. McGuigan KG, Samaiyar P, du Preez M, Conroy RM. High Compliance Randomized Controlled Field Trial of Solar Disinfection of Drinking Water and Its Impact on Childhood Diarrhea in Rural Cambodia. Environmental Science & Technology. 2011;45(18):7862-7.

28. Kundu DK, Gupta A, Mol APJ, Nasreen M. Understanding social acceptability of arsenic-safe technologies in rural Bangladesh: a user-oriented analysis. Water Policy. 2016;18(2):318-34.

29. Harrison BL, Ogara C, Gladstone M, Carrol ED, Dusabe-Richards J, Medina-Lara A, et al. "We have to clean ourselves to ensure that our children are healthy and beautiful": findings from a qualitative assessment of a hand hygiene poster in rural Uganda. Bmc Public Health. 2019;19.

30. Firth J, Balraj V, Muliyil J, Roy S, Rani LM, Chandresekhar R, et al. Point-of-use interventions to decrease contamination of drinking water: a randomized, controlled pilot study on efficacy, effectiveness, and acceptability of closed containers, Moringa oleifera, and in-home chlorination in rural South India. The American journal of tropical medicine and hygiene. 2010;82(5):759-65.

31. Rajasingham A, Hardy C, Kamwaga S, Sebunya K, Massa K, Mulungu J, et al. Evaluation of an Emergency Bulk Chlorination Project Targeting Drinking Water Vendors in Cholera-Affected Wards of Dar es Salaam and Morogoro, Tanzania. American Journal of Tropical Medicine and Hygiene. 2019;100(6):1335-41.

32. Stone MA, Ndagijimana H. Educational intervention to reduce disease related to sub-optimal basic hygiene in Rwanda: initial evaluation and feasibility study. Pilot Feasibility Stud. 2018;4:4.

33. Biran A, Danquah L, Chunga J, Schmidt WP, Holm R, Itimu-Phiri A, et al. A Cluster-Randomized Trial to Evaluate the Impact of an Inclusive, Community-Led Total Sanitation Intervention on Sanitation Access for People with Disabilities in Malawi. American Journal of Tropical Medicine and Hygiene. 2018;98(4):984-94.

34. Francis MR, Nagarajan G, Sarkar R, Mohan VR, Kang G, Balraj V. Perception of drinking water safety and factors influencing acceptance and sustainability of a water quality intervention in rural southern India. BMC Public Health. 2015;15(1).

35. Habib MA, Soofi S, Sadiq K, Samejo T, Hussain M, Mirani M, et al. A study to evaluate the acceptability, feasibility and impact of packaged interventions ("Diarrhea Pack") for prevention and treatment of childhood diarrhea in rural Pakistan. Bmc Public Health. 2013;13.

36. Hussain F, Luby SP, Unicomb L, Leontsini E, Naushin T, Buckland AJ, et al. Assessment of the Acceptability and Feasibility of Child Potties for Safe Child Feces Disposal in Rural Bangladesh. American Journal of Tropical Medicine and Hygiene. 2017;97(2):469-76.

**List of Legends**

Table A. The majority of included WASH acceptability studies did not provide an explicit definition of acceptability. To facilitate comparisons between the included articles, we developed a categorisation system for the acceptability measures used across the 36 papers. Acceptability measures were grouped based on similar characteristics. The article definition categorisations were independently reviewed by two authors (RH, SYO). Definitions with quotations “ ” were supplied by the study authors, all others are implicit definitions.
